# Efficacy of vitrectomy with air tamponade for rhegmatogenous retinal detachment: a prospective study

**DOI:** 10.1038/s41598-023-37693-x

**Published:** 2023-07-04

**Authors:** Katsuhiro Nishi, Madoka Nakamura, Koichi Nishitsuka

**Affiliations:** Department of Ophthalmology and Visual Sciences, Yamagata University Faculty of Medicine, 2-2-2 Iida-Nishi, Yamagata, Yamagata 990-9585 Japan

**Keywords:** Diseases, Medical research

## Abstract

Rhegmatogenous retinal detachment (RRD) causes a permanent decrease in visual acuity and visual field. During pars plana vitrectomy (PPV) for RRD, long acting gas have been selected for tamponade because gas stays in the eye for a long time. Recently, several studies have showed the efficacy of air tamponade for RRD treatment. Few prospective studies have analyzed the efficacy of air tamponade. We registered 194 eyes from 190 patients who consented to a prospective study of PPV with air tamponade for RRD by a single surgeon from June 2019 to November 2022. These patients were all treated with air tamponade without silicone oil and were followed for > 3 months postoperatively. Primary success rates were 97.9% (190/194) in total cases, with no discernible difference between the uncomplicated (100%:87/87) and complicated (96.3%: 103/107) RRD groups (P = 0.13). There was no considerable difference in primary success rate between upper break (97.9%:143/146) and lower break cases (97.9%:47/48). Proliferative vitreoretinopathy (PVR) grade C was associated with initial failure by multivariate analysis (P = 0.00003). Air tamponade has a sufficient therapeutic effect in cases of RRD less than PVR grade C, regardless of the location of the retinal tear.

## Introduction

The incidence of rhegmatogenous retinal detachment (RRD) has been reported to be between 6.3 and 17.9 per 100,000 people^1^. This ophthalmological disease causes a permanent decrease in visual acuity and visual field, potentially leading to vision loss^2^. Proliferative vitreoretinopathy (PVR) is an intractable complication of RRD^3^. Surgeons determine RRD procedures based on the location of the retinal breaks and disease severity. These surgical procedures include pars plana vitrectomy (PPV), scleral buckling (SB), and pneumatic retinopexy^4^. Some patients with RRD may benefit from PPV plus SB, but it depends on disease severity. A study conducted by the Japan Retinal Detachment Registry Group has indicated that the most common surgical procedure for RRD is PPV (77.8%)^5,6^.

During PPV for RRD, an intraocular tamponade is inserted at the end of surgery and patients are placed in the prone position. Air, long-lasting gases such as sulfur hexafluoride (SF6) and octafluoropropane (C3F8), and silicone oil are all options for intraocular tamponade agents. Surgeons select agents based on RRD severity. Most surgeons have used SF6 and C3F8 because these remain in the eye longer than air, leading to favorable outcomes for retinal reattachment^7,8^. However, SF6 and C3F8 have some drawbacks, such as a long-term prone position and increased intraocular pressure, which could be increased after surgery if the optimal density is not achieved. Silicone oil tamponade has frequently been used for patients with severe RRD and concomitant PVR. However, silicone oil does not disappear spontaneously, a second surgery is required to remove it.

Recently, some retrospective studies have reported the efficacy of air tamponade during PPV for RRD^9–11^. Because the duration of air within the eye after PPV is short, air tamponade may benefit patients by reducing the time spent in the prone position and the risk of increased intraocular pressure. However, due to the short duration of air in the eye, retinal detachment can occur again after PPV, even in patients with mild-to-moderate RRD. There are no clear standards for selecting an air tamponade according to severity; thus, surgeons at each medical institution determine which tamponade agent is used.

We previously conducted a retrospective study and reported the selection criteria for air tamponade ^12^. The retrospective study excluded patients with RRD treated with PPV plus SB and those who met the exclusion criteria (i.e., more than 2 weeks after onset, giant retinal tears, history of cataract surgery complication, high myopia, and PVR classified as grade C or higher). As a result, 294 eyes with RRD were included (156 eyes in the air group and 138 eyes in the SF6 group), and no difference was observed in the primary success rate between the two groups.

The present study aimed to prospectively evaluate the usefulness of the selection criteria for air tamponade and the efficacy of vitrectomy using air tamponade for patients with RRD.

## Results

This study included 194 eyes of 190 patients with RRD who underwent PPV with air tamponade (Supplementary Information). Table 1 shows the patients’ characteristics. A total of 28 eyes (14.4%) had pseudophakia before surgery. The history of complications following cataract surgery was unknown.

**Table 1:** Patient’s characteristics.

Patient’s characteristics	n = 194
Age (years), mean ± SD	61.4 ± 8.6
Sex	
Female	49 (25.2%)
Male	145 (74.7%)
Interval from onset to surgery (days), mean ± SD	12.7 ± 9.0
History of atopic dermatitis	0 (0%)
Axial length (mm), mean ± SD	25.5 ± 1.4
Quadrant of retinal detachment	
1	52 (26.8%)
2	107 (55.2%)
3	23 (11.8%)
4	12 (6.2%)
Number of breaks	
1	85 (43.8%)
2	52 (26.8%)
3+	57 (29.4%)
Type of the break	
Tear	169 (87.1%)
Hole	25 (12.9%)
Location of break	
Upper break	146 (75.2%)
Lower break	48 (24.8%)
Giant retinal break	2 (1.0%)
PVR	
N/grade A	160 (82.5%)
Grade B	18 (9.3%)
Grade C	16 (8.2%)
Choroidal detachment	6 (3.0%)
Macular detachment	97 (50.0%)
Lens status	
Phakia	166 (85.6%)
Pseudophakia	28 (14.4%)

*SD* standard deviation, *PVR* proliferative vitreoretinopathy, *N/grade A* none or grade A.

Table 2 shows the surgical results. The primary success rate was 97.9% (190/194). The incidence of postoperative ocular hypertension was 23.2% (45/194). Air half-life was 4.8 ± 0.6 days. Because the postoperative patients were examined daily and released from the prone position when the intraocular air was reduced by half, the average duration of the prone position was also 4.8 ± 0.6 days.

**Table 2:** Surgical results.

Primary success	190 (97.9%)
Operating time (minutes), mean ± SD	43.9 ± 8.7
Iatrogenic retinal hole	35 (18.0%)
Postoperative ocular hypertension	45 (23.2%)
Air half-life (day), mean ± SD	4.8 ± 0.6

*SD* standard deviation.

Table 3 shows details of the primary success rate. The primary success rate was 100% (87/87) in uncomplicated RRD and 96.3% (103/107) in complicated RRD, with no significant difference between the two groups (P = 0.13). The primary success rate by location of retinal breaks was 97.9% (143/146) in upper breaks and 97.9% (47/48) in lower breaks, with no significant difference between the two groups (P = 0.99). The primary success rate by lens status was significantly lower in pseudophakia [92.9% (26/28)] than in phakia [98.8% (164/166); all patients underwent cataract surgery; P = 0.008]. The primary success rate in the RD quadrant decreased as the area of RD widened (P = 0.008). The primary success rate was 98.2% (54/55) in an axial length of > 26.5 mm and 97.8% (136/139) in an axial length of 26.5 mm, with no significant difference between the two groups (P = 0.62). The primary success rate decreased as the severity of PVR increased, and a significant decrease was observed in PVR classified as grade C (P = 0.0000009). The primary success rate was significantly lower [83.3% (5/6)] in patients with choroidal detachment (P = 0.011).

**Table 3:** Details of the primary success rate.

			P value
Severity of RRD			0.13^a^
Uncomplicated RRD	100% (87/87)		
Complicated RRD	96.3% (103/107)		
Location of break			0.99^a^
Upper break	97.9% (143/146)		
Lower break	97.9% (47/48)		
Lens status			0.041^a^
Phakia	98.8% (164/166)		
Pseudophakia	92.9% (26/28)		
Quadrant of RD			0.008^b^
1	100% (52/52)		
2	99.1% (106/107)		
3	95.7% (22/23)		
4	83.3% (10/12)		
Axial length			0.62^a^
> 26.5	98.2% (54/55)		
26.5 ≥	97.8% (136/139)		
Giant retinal break	100% (2/2)		–
PVR			
N/grade A	100% (160/160)		
Grade B	94.4% (17/18)	Vs grade N/A	0.10^a^
Grade C	81.2% (13/16)	Vs grade N/A + B	< 0.001^a^
Choroidal detachment	83.3% (5/6)		0.011^a^

*RRD* rhegmatogenous retinal detachment, *PVR* proliferative vitreoretinopathy, *N/grade A* none or grade A.

^a^*P* values were calculated with Fisher’s exact test.

^b^*P* values were calculated with Mann–Whitney U test.

To identify factors contributing to the primary success rate, multivariate analyses were conducted using factors found to be statistically significant in the univariate analysis. The results are shown in Table 4. PVR grade C was negatively correlated with the primary success rate (P = 0.00003, OR = −0.155, 95% CI −0.23 to 0.0084).

**Table 4:** Multivariate analysis of primary success.

	Univariate analysis	Multivariate analysis		
	P value	OR	95% CI	P value
Choroidal detachment	0.011	−0.068	−0.19 to 0.50	0.26
Pseudophakia	0.041	−0.037	−0.09 to 0.02	0.18
Quadrant of retinal detachment	0.008	−0.017	−0.04 to 0.01	0.22
PVR grade C	< 0.001	−0.155	−0.23 to −0.084	< 0.001

*PVR* proliferative vitreoretinopathy, *OR* odds ratio, *95%CI* 95% confidence limits.

In total, four eyes of four patients were not responsive to primary PPV with air tamponade. The breakdown of these cases was total retinal detachment in one eye (PVR grade C-1), an axial length of > 26.5 mm in one eye, a history of SB on the temporal side of the retina 35 years ago in one eye, and RRD > 2 weeks since onset, choroidal detachment, and total retinal detachment in one eye (PVR grade B).

A surgeon determined the use of silicone oil tamponade during the primary vitrectomy for 16 eyes of 16 patients, although they were not included in this study. The breakdown of these cases was PVR grade C-2 or higher in 14 eyes, acute retinal necrosis in one eye, and unable to maintain the prone position for a long period of time due to high-grade obesity in one eye (PVR grade B). The 14 eyes were determined preoperatively to have PVR grade C-1 but PVR grade C-2 or higher intraoperatively, so silicone oil was injected.

## Discussion

This study included 194 eyes of 190 patients with RRD who underwent PPV with air tamponade and were followed up for more than 3 months after surgery. The primary success rate was 97.9% (190/194), with no significant difference between uncomplicated RRD and complicated RRD as well as between upper and lower breaks. PVR grade C was the only factor negatively correlated with the primary success rate. Patients with RRD who did not require silicone oil tamponade responded well to PPV with air tamponade.

RRD was divided into two conditions: uncomplicated RRD and complicated RRD. The success rate was favorable at 100% (87/87) in uncomplicated RRD and 96.3% (103/107) in complicated RRD, with no significant difference between the two groups. Uncomplicated RRD met the selection criteria for air tamponade during PPV for RRD, which was previously proposed by the authors^12^. This demonstrated the efficacy of the selection criteria for air tamponade in the present study. Furthermore, the present study showed that air tamponade was useful for patients with PVR of grade B or lower even if the severity of PVR was beyond the above-mentioned selection criteria for air tamponade. Li et al. retrospectively evaluated 59 eyes of 59 patients with RRD who underwent PPV with air tamponade. They reported that the primary anatomical rate was 94.9% (56/59) and the final anatomical success rate was 98.3% (58/59). That study included two eyes of two patients with PVR grade C-1 (3.4%). One of the patients who did not respond to the primary PPV had postoperative progression of PVR. Another patient experienced RRD associated with a macular hole in high myopia. They concluded that PPV with air tamponade may be effective in treating selected cases^9^. The present study used air tamponade for patients with PVR grade C-1 or lower (i.e., subretinal strands developed but retinal fold formation was limited). For patients with PVR grade C-1 or higher, a surgeon determined whether silicone oil injection was needed or not during surgery. The results of the multivariate analysis of the primary success rate showed that air tamponade was not recommended for patients with RRD of PVR grade C or higher.

A previous study reported that the anatomic success rate of the primary vitrectomy for RRD with lower breaks was low ^8^. Tan et al. reported that RRD patients with lower breaks who underwent PPV had significantly lower primary success rates when using air tamponade compared with gas tamponade (69.6% vs 84.7%, P = 0.009). They concluded that air tamponade should only be used in RRD that is restricted to upper breaks ^11^. Zhou et al. prospectively evaluated RRD patients with lower breaks who underwent PPV. The patients were randomly assigned to the air tamponade group or the C3F8 tamponade group. There was no significant difference in the success rate between the two groups, indicating that air tamponade was effective for RRD with lower breaks ^7^.

According to a study conducted by Singh et al., the overall success rate in treating RRD patients with superior, inferior, and multiple breaks who underwent PPV was 88.5% (146/165 eyes) with air tamponade and 80.3% (57/71 eyes) with SF6 20% tamponade. Preoperative characteristics were almost similar between the two groups. They reported that PPV with air tamponade was effective for RRD regardless of the location of the retinal breaks ^13^. Tetsumoto et al. retrospectively evaluated patients with RRD who underwent 27-gauge PPV. The patients were divided into two groups (Group A: 20% SF6 was applied to 35 eyes; Group B: air was used in 35 eyes). There was no significant difference in the initial and final anatomical success rates between the two groups (initial: Group A 97.1% vs. Group B 94.3%, P = 1; final: Group A 100% vs. Group B 100%, P = 1) ^14^. The present study prospectively evaluated the primary success rate based on the location of retinal breaks (i.e., upper and lower breaks). The results were favorable, with no significant difference between the two groups, demonstrating that air tamponade was effective even for lower breaks if the severity of PVR was grade B or lower. This result was thought to be due to the vitreous being removed sufficiently up to the vitreo-retinal angle formed by the retina and peripheral posterior vitreous detachment near the vitreous base to eliminate the traction caused by the vitreous ^15–17^.

The incidence of postoperative ocular hypertension was 23.2% (45/194) in the present study. However, it was 2% with air tamponade and 5% with SF6 tamponade in a study conducted by Uemura et al. ^10^. A possible explanation for this difference may be a difference in the definition. Postoperative ocular hypertension was defined as > 25 mmHg in that study, and it was defined as ≥ 22 mmHg in the present study. The incidence of postoperative ocular hypertension was similar between a retrospective study ^12^ (19.9%, air tamponade group) and the present study. Unlike long-acting gases, air does not inflate in the eye, possibly leading to a lower rate of postoperative ocular hypertension.

The duration of the half-life of air differs from study to study; it was 4.8 ± 0.6 days in the present study, 1.6 days in a study conducted by Thompson in 1898 ^18^, and approximately 3.3 days in a study conducted by Lee et al. in 2020 ^19^. Sufficient shaving of the peripheral vitreous was associated with an increased amount of air injection, leading to the extension of the half-life of air. In the present study, peripheral vitreous shaving was performed up to the vireo-retinal angle formed by the retina and peripheral posterior vitreous detachment near the vitreous base ^15–17^. As a result, the amount of air injected into the eye may have increased in the present study as compared with previous studies. Furthermore, compared with cases without sutures, instances of air leakage may have been fewer in patients in the present study because scleral ports were closed with absorbable sutures at the end of surgery. A retrospective study that used the same surgical procedure as the present study showed that the half-life of air was 3.97 ± 0.87 days ^12^. The results were similar to those of the present study. Adhesion around the retinal break occurred within approximately 4 days of the air half-life, as strong adhesion between the retinal pigment epithelium and the sensory retina could occur within 24 h after photocoagulation ^20,21^.

The need for a strict prone position after surgery has been debated because the efficacy of intraocular gas tamponade is low in RRD with lower breaks. Martinez et al. reported the efficacy of 24 h of prone positioning in the management of lower breaks in patients with pseudophakic RRD ^22^. They subsequently conducted another study and reported that the prone position is not necessary to achieve retinal reattachment in the same population ^23^. In a study conducted by Uemura et al., patients were instructed to maintain the prone position for 1 h after surgery and the lateral recumbent position for the next 24 h ^10^. These studies involved patients with uncomplicated RRD. The present study included patients with complicated RRD with several locations of retinal breaks. They were instructed to maintain the prone position until the intraocular air concentration was reduced by half. The present study did not adjust the duration of the prone position according to the location of retinal breaks. Therefore, whether differences in the duration of the prone position affect the primary success rate is unknown. This is an issue that needs more research and discussion.

The strength of the present study is that it is prospective in nature. A single surgeon with sufficient skill performed vitrectomy on patients with RRD, which minimized the bias related to surgical outcomes. Most previous studies retrospectively evaluated the efficacy of vitrectomy for RRD performed by several surgeons. Thus, differences in skill and decision-making methods might have affected the surgical outcomes. There were also several limitations in this study. The fact that the vitrectomy was performed by a single surgeon with sufficient skill to perform vitrectomy on patients with RRD minimized bias in this study but prevented generalization of the surgical outcomes. The postoperative follow-up period was only 3 months, which may have been too short to adequately assess postoperative complications. The present study is a single-center study with a small sample size. A multicenter, prospective study involving more patients will be required. Furthermore, to evaluate the usefulness of the results of the present study, a multicenter, prospective study in which several surgeons perform vitrectomy using the modified criteria of performing air tamponade for PVR grade B or less will be needed. In addition, a follow-up period of at least 6 months postoperatively would be necessary to fully evaluate postoperative complications ^10,14^. The surgical outcomes of pneumatic retinopexy with air tamponade are unknown because it has not been performed yet in our institution.

PPV with air tamponade for RRD is associated with favorable surgical outcomes both in uncomplicated RRD and complicated RRD regardless of the location of retinal breaks, except for patients with severe RRD who are classified as PVR grade C and require silicone oil tamponade. A multicenter study involving more patients will be required.

## Methods

After informed consent was obtained, 210 eyes of 206 patients were subjected to primary vitrectomy with air tamponade at Yamagata University Faculty of Medicine between June 19, 2019, and November 4, 2022. A total of 16 eyes of 16 patients were diagnosed with severe RRD during surgery, necessitating the use of silicone oil tamponade. This study included 194 eyes of 190 patients who were followed up for more than 3 months after vitrectomy with air tamponade. Inclusion criteria for this study were patients with RRD who underwent vitrectomy with air tamponade rather than scleral buckling or pneumatic retinopexy. At our institution, scleral buckling was the first choice for juvenile rhegmatogenous retinal detachment. Exclusion criteria were cases of PVR grade C-2 or higher. This prospective study was approved by the Ethics Committee of Yamagata University School of Medicine and conducted in accordance with the Declaration of Helsinki. This study evaluated the following: age, sex, interval from onset to surgery (days), history of atopic dermatitis, traumatic retinal detachment, axial length (mm), quadrant of retinal detachment, number of breaks, type of break, location of break (defined the 4–8 o’ clock quadrant as the lower break and the other quadrants as the upper break ^10^), giant retinal break, PVR (N/grade A, B, C) ^3^, choroidal detachment, macular detachment, lens status (Phakia/Pseudophakia), iatrogenic retinal hole, operating time (minutes), primary success rate, postoperative ocular hypertension (≥ 22 mmHg), air half-life (days).

RRD > 2 weeks since onset, giant retinal tears, history of complications following cataract surgery (posterior capsule rupture), high myopia (axial length > 26.5 mm or the spherical equivalent < − 6.0D), and severe proliferative vitreoretinopathy (PVR) grade C were defined as complicated RRD and others were defined as uncomplicated RRD.

### Surgical procedures

A single surgeon (K.N) performed 25-gauge (G) PPV using an EVA vitrectomy system (DORC, Zuidland, The Netherlands), a wide-angle noncontact viewing system (Resight; Carl Zeiss Meditec AG, Jena, Germany), and a RESCAN 700 surgical microscope (Carl Zeiss Meditec AG, Jena, Germany). Cataract surgery was concurrently performed on all phakic eyes. After a 3-port core vitrectomy was performed, vitreous gel was visualized with an injection of triamcinolone acetonide (MaQaid, Wakamoto Pharmaceutical, Tokyo, Japan). The peripheral vitreous gel was shaved with a scleral indentation under chandelier lighting. Peripheral vitreous shaving was performed up to the vireo-retinal angle formed by the retina and peripheral posterior vitreous detachment near the vitreous base ^15–17^. If patients had a subretinal strand, it was not removed. Internal drainage of subretinal fluid was performed through a pre-existing retinal break rather than creating an intentional retinal break. Then, a fluid-air exchange was performed. Photocoagulation (Solitaire; Ellex, Australia) was applied in three lines to the surroundings of all retinal breaks under the condition that power was 150 mW and duration time was 0.3 s. No concomitant use of SB or 360-degree retinal photocoagulation was performed. Scleral port areas were closed with 8-0 Polysorb. Air was additionally injected into the vitreous cavity through a filter if the adjustment of intraocular pressure was needed before completing surgery. Patients were instructed to remain in the prone position following surgery until the intraocular air concentration was reduced to half. Subsequently, all patients were permitted to assume any position except the supine position.

### Statistical analysis

Fisher’s exact test and Mann–Whitney U test were used, and the significance level was set at P < 0.05. In terms of the primary success rate, multivariate analyses were conducted using factors found to be statistically significant in the univariate analysis. Statistical analyses were performed using PASW Statistics 18 (SPSS Inc., Chicago, IL, USA).

## Supplementary Information

Supplementary Information.

## Data Availability

All relevant data are within the paper and its Supplementary Information files (Raw data).

## References

[1] Mitry D, Charteris DG, Fleck BW, Campbell H, Singh J (2009). The epidemiology of rhegmatogenous retinal detachment: Geographical variation and clinical associations. Br. J. Ophthalmol..

[2] D'Amico DJ (2008). Primary retinal detachment. N. Engl. J. Med..

[3] Machemer R (1991). An updated classification of retinal detachment with proliferative vitreoretinopathy. Am. J. Ophthalmol..

[4] Nemet A, Moshiri A, Yiu G, Loewenstein A, Moisseiev E (2017). A Review of innovations in rhegmatogenous retinal detachment surgical techniques. J. Ophthalmol..

[5] Sakamoto T (2020). Japan-Retinal Detachment Registry Report I: Preoperative findings in eyes with primary retinal detachment. Jpn. J. Ophthalmol..

[6] Nishitsuka K (2020). Preoperative factors to select vitrectomy or scleral buckling for retinal detachment in microincision vitrectomy era. Graefes Arch. Clin. Exp. Ophthalmol..

[7] Zhou C, Qiu Q, Zheng Z (2015). Air versus gas tamponade in rhegmatogenous retinal detachment with inferior breaks after 23-gauge pars plana vitrectomy. Retina.

[8] Goto T, Nakagomi T, Iijima H (2013). A comparison of the anatomic successes of primary vitrectomy for rhegmatogenous retinal detachment with superior and inferior breaks. Acta Ophthalmol..

[9] Li Y, Cheung N, Jia L, Zhang H, Liu N (2020). Surgical outcomes of 25-gauge pars plana vitrectomy using air as an internal tamponade for primary rhegmatogenous retinal detachment. Retina.

[10] Uemura A (2022). Air versus sulfur hexafluoride gas tamponade in vitrectomy for uncomplicated retinal detachment with inferior breaks. Retina.

[11] Tan HS, Oberstein SY, Mura M, Bijl HM (2013). Air versus gas tamponade in retinal detachment surgery. Br. J. Ophthalmol..

[12] Nakamura M, Nishi K, Nishitsuka K (2022). Selection criteria for air tamponade during vitrectomy for rhegmatogenous retinal detachment. Clin. Ophthalmol..

[13] Singh A, Boustani G, Michez M, Bali E (2021). Routine use of air tamponade in pars plana vitrectomy for primary rhegmatogenous retinal detachment repair. Ophthalmologica.

[14] Tetsumoto A (2020). The comparison of the surgical outcome of 27-gauge pars plana vitrectomy for primary rhegmatogenous retinal detachment between air and SF6 gas tamponade. Eye (Lond).

[15] Nishitsuka K (2021). Surgical outcomes of rhegmatogenous retinal detachment with different peripheral vitreous-shaving procedures. Clin. Ophthalmol..

[16] Nishitsuka K, Nishi K, Namba H, Kaneko Y, Yamashita H (2021). Quantification of the peripheral vitreous after vitreous shaving using intraoperative optical coherence tomography. BMJ Open Ophthalmol..

[17] Nishitsuka K, Nishi K, Namba H, Kaneko Y, Yamashita H (2018). Intraoperative optical coherence tomography imaging of the peripheral vitreous and retina. Retina.

[18] Thompson JT (1989). Kinetics of intraocular gases: Disappearance of air, sulfur hexafluoride, and perfluoropropane after pars plana vitrectomy. Arch. Ophthalmol. (Chicago Ill, 1960).

[19] Lee JJ (2020). Duration of room air tamponade after vitrectomy. Jpn. J. Ophthalmol..

[20] Kita M, Negi A, Kawano S, Honda Y (1991). Photothermal, cryogenic, and diathermic effects of retinal adhesive force in vivo. Retina.

[21] Yoon YH, Marmor MF (1988). Rapid enhancement of retinal adhesion by laser photocoagulation. Ophthalmology.

[22] Martínez-Castillo V (2005). Management of inferior breaks in pseudophakic rhegmatogenous retinal detachment with pars plana vitrectomy and air. Arch. Ophthalmol..

[23] Martínez-Castillo V, Boixadera A, Verdugo A, García-Arumí J (2005). Pars plana vitrectomy alone for the management of inferior breaks in pseudophakic retinal detachment without facedown position. Ophthalmology.

